# Drug Therapy in Cognitive Disorders and Its Effects on Oral Health

**DOI:** 10.7759/cureus.27194

**Published:** 2022-07-24

**Authors:** Syed Ershad Ahmed, Rizwana Begum, Aparna S Kumar, Arun M, Vaishnavi R, Vinith I

**Affiliations:** 1 Prosthodontics, Sri Ramakrishna Dental College and Hospital, Coimbatore, IND; 2 Oral Medicine, Vinayaka Missions Research Foundation (VMRF) Deemed to be University, Salem, IND; 3 Anatomy, Sri Ramakrishna Dental College and Hospital, Coimbatore, IND; 4 Prosthodontics and Crown and Bridge, Sri Ramakrishna Dental College and Hospital, Coimbatore, IND; 5 Dentistry, Sri Ramakrishna Dental College and Hospital, Coimbatore, IND

**Keywords:** dementia, alzheimer’s disease, cognitive dysfunctions, dental drugs, pharmacological agents

## Abstract

Dementia and Alzheimer’s disease are the two most characteristic cognitive disorders presenting numerous cognitive dysfunctions such as memory loss, functional impairment, speech impairment, and orientation problems. In India, there is an increased risk in the elderly population leading to the prevalence of Alzheimer's and dementia-related diseases. Therefore, it is not only general health care practitioners but also oral health care providers also play a major role in rehabilitating and treating this type of patient. So, it is necessary for oral health care providers to understand the pharmacologic agents used for the management of Alzheimer’s and dementia-related diseases. This article gives an insight into the management of dementia and Alzheimer’s disease and also an update on the drug therapies for AD and outlines their implications on oral health.

## Introduction and background

Dementia can be defined as a neurodegenerative disease that causes acquired, progressive deterioration in cognitive abilities that impairs the successful performance of activities of daily living [1]. Alzheimer's disease (AD) is the type of dementia that accounts for 60%-70% of dementia cases [2]. It leads to loss of memory and cognitive functioning in a progressive manner thus causing deficiencies in language and visuospatial skills. These deficiencies are often accompanied by apathy, aggressiveness, and depression [3].

In India, the number of elderly people is expected to rise drastically due to increased life expectancy caused by the newer technologies in the medical field. By 2100, in India, the number of elderly people will increase by one elderly for every three working age population [4]. Around 3.7 million people in the Indian population will with dementia and dementia-related problems in 2010 and studies show that these are expected to double by 2030, having a wide impact in developing countries like India [5].

To design protective and preventing interventions for dementia, it is always necessary to understand the risk factors of dementia. Risk factors can be grouped as modifiable and non-modifiable factors. Nonmodifiable factors are classified as age, family history, apoE4 allele, female sex, depression, head trauma, mutation on 1,14,21 chromosome, and down’s syndrome whereas the modifiable factors would be any vascular disease, hypertension, diabetes, dyslipidemia, nutritional deficiency (B vitamins), smoking, alcohol, obesity, diet [5]. Senile plaques and neurofibrillary tangles are the neuropathological features seen in AD. The deposition of extracellular plaques of insoluble β-amyloid peptide and neurofibrillary tangles are the main characteristic features of AD. According to the theories of pathogenesis, the amyloid cascade theory suggests that the cerebral build-up of Aβ peptide causes an alteration in the production and clearance of protein, along with the formation of neurofibrillary tangles is the main cause of the disease [3,6]. Pathological examination in AD shows that there is degeneration in cholinergic neuron-rich regions, such as the nucleus basalis of Meynert, frontal cortex, anterior cingulate cortex, and posterior cingulate cortex [2].

Studies in the literature have assessed the importance of dentition and chewing ability on cognitive functioning. A study conducted by Seraj et al. concluded that people with a fewer number of teeth had impaired chewing ability which led to decreased cognitive function [7]. A study by Fanny et al. suggested that chewing efficiency had a significant role in enhancing the cognitive functioning of the patient [8]. Campos et al. in their study revealed that patients with mild Alzheimer’s disease had lower masticatory performance and also low Mini-mental state examination (MMSE) values. According to the literature, low masticatory performance leads to decreased brain memory stimulation [9-11]. So, the aim of the present paper is to provide a narrative review of the effects of cognitive disorders on oral health and the interactions of the anti-cognitive disorder drugs on oral health and dental drug therapy.

## Review

Effects of cognitive disorders on oral health, masticatory efficiency

Cognitive disorders mostly affect the elderly population, individuals with cognitive dysfunctions struggled to perform basic daily living activities and also failed in maintaining good oral health. Cognitive Impairment is often followed by deterioration of oral health which is often reflected in a progression of periodontal disease, caries, and tooth loss. People with cognitive impairment do not have the capacity to understand the problems and changes associated with the condition and also there is a lack of interest in maintaining good oral care by brushing and flossing. Studies have shown that the number of cases of caries was significantly higher in patients with dementia than with other patients without dementia [12,13]. These findings were associated with gingival problems leading to the substantial accumulation of plaque with increased gingival bleeding, thus contributing to low mucosal health [14].

Edentulism is considered to be a main problem in the elderly the World Health Organization estimated the prevalence of edentulism among 65-74-year olds in India at 19 % [15]. Caries and periodontitis are the two main causes of tooth loss among other factors such as poor oral hygiene and low socioeconomic status. Tooth loss is a marker of pathologic oral inflammatory conditions and several cross-sectional studies have shown the relationship between tooth loss and cognitive impairment [7,8]. A study conducted by Ranjan et al 2019 has shown that a number of dentitions and MMSE values have a direct relation to the Indian population. Low MMSE scores were associated with patients who had fewer dentitions. This was attributed to many factors such as the socioeconomic status, school education, and marital status of the individuals [16].

It has been reported that mastication and cognition are interrelated. Chewing enhances the blood flow thereby stimulating the perfusion rate of the oxygen in the brain. This mechanism has been proved to be a protective factor against dementia and neurodegenerative diseases [17]. This hypothesis is also supported by various studies which state that patients with more teeth have a greater masticatory efficiency and they tend to have a lower rate of dementia and less cognitive dysfunction [18]. Chewing improves the neuronal centers and activities in the brain related to biting force. This performance is because of lesser stimulation of memory areas in the brain [19,20]. Mastication stimulates prefrontal activation, thereby reducing prefrontal depression. In patients with loss of teeth or missing teeth, this prefrontal activation through mastication is absent. This is also commonly seen in AD and dementia-related patients as there is decreased masticatory efficiency. Therefore, it is proved by various studies the importance of replacing the missing teeth with prostheses/dentures, which increases the chewing pattern and thereby promotes prefrontal activities and increased cognition [20].

Medications/therapies involved in cognitive disorders

Early diagnosing of AD and other dementia related problems is very important before the life - altering features starts to appear. Both pharmacologic and non-pharmacologic treatment methods should be considered for AD and dementia related problems. Non-pharmacologic method includes having an environment which is familiar and harmless to the patient and also removing the stimulus thereby moderating effects of the disease but these approaches are mostly seem to be unsuccessful [21].

The primary objective of the pharmacologic therapy is to balance and reduce the cognitive, functional, and behavioral symptoms. These medications are to treat the symptomatic effects rather than the pathology itself [22]. Current line of drug regimen includes cholinesterase inhibitors (ChEIs) and N-methyl-D-aspartate receptor antagonists as the first line of medications in treating AD and dementia related problems. To treat the non-cognitive psychiatric symptoms, adjunctive drugs such as antipsychotics, antidepressants, and anxiolytics are used [23]. Recently researchers have developed various new line of drugs and interventions, to relieve behavioral psychological symptoms (Table 1).

**Table 1:** Mechanism of action of drugs used in cognitive disorders

S.NO	CLASS OF DRUGS	MECHANISM OF ACTION
1.	Cholinesterase inhibitors Donepezil	Availability of acetylcholine is increased ; blood flow is enhanced
	Galantamine	
	Rivastigmine	
2	N-methyl-D-aspartate (NMDA) receptor antagonists Memantine	Glutamate excitotoxicity is prevented by antagonising NMDA receptor activity
3.	Anti-amyloid therapy Tarenflurbil	Accumulation of Aβ42 amyloid plaques is reduced
4.	Anti-tau therapy	Level of aggregated tau proteins is decreased to lessen tau-related neuronal damage
	TRx0237 (LMTX)	
	AADvac1	Inhibits tau aggregation and reduces the development of neurofibrillary tangles
	Zagotenemab (LY3303560)	Anti-tau antibody developed to capture and neutralize tau aggregate
5.	Anti -neuroinflammation therapy Azeliragon	Antagonist of the receptor for advanced glycation end products (RAGE). regulates transport of circulating plasma Aβ to the brain, inflammatory process, oxidation stress, and cerebral blood flow
6.	Neuroprotection	Reduces synaptic glutamate level and increase the synaptic glutamate absorption.
	BHV-4157 (troriluzole)	
	Ginkgo biloba extract (GBE)	Anti-oxidative; weak anti-platelet activity; blood flow enhancer
7.	Cognitive enhancers RVT-101 (intepirdine)	Postsynaptic 5- hydroxytryptamine (5-HT) 6 receptor antagonist. Helps in maintaining the balance between excitatory and inhibitory signals by regulating the GABA and glutamate levels in different neuronal junctions

The dosage of drugs associated with Alzheimer’s disease and cognitive disorders are given in Table 2.

**Table 2:** Oral dosage of drugs used in Alzheimer disease and other cognitive disorders

S.NO	DRUGS	ORAL DOSAGE
1.	Cholinesterase inhibitors	
	Donepezil Galantamine Rivastigmine	5-10 mg daily 8-24 mg daily 1.5-6 mg twice daily
2.	N-methyl-D-aspartate receptor Antagonists	
	Memantine	5-10 mg twice daily
3.	Tricyclic antidepressants	
	Desipramine Amitriptyline	10-150 mg daily 10-50 mg daily
4.	Typical antipsychotics	
	Haloperidol	0.25-5 mg every 6 h
5.	Atypical antipsychotics	
	Risperidone Olanzapine	0.25-2 mg daily 2.5-10 mg daily
6.	Benzodiazepines	
	Lorazepam Oxazepam Triazolam	1-2 mg daily 10-60 mg daily 0.125-0.25 mg daily

Adverse effects associated with anti-cognitive disorder drugs

There is an increased risks for adverse drug effects with patients with AD and other cognitive disorders as they are on multiple drug medication. These adverse effects associated with drugs of cognitive disorders are commonly due to their mechanism of action. These effects are seen affecting the general health and also the oral health of the patient (Table 3) [23].

**Table 3:** Adverse effects affecting the general health associated with anti-Alzheimer and cognitive drugs

S. no	Drugs	Adverse effects
1.	Cholinesterase inhibitors	1. nausea, vomiting, diarrhea, anorexia, abdominal pain 2. headache, dizziness, fatigue, anxiety, syncope 3.myalgia, muscle cramps
2.	N-methyl-D-aspartate receptor Antagonists	1. dizziness, confusion, headache, hallucinations 2. coughing, vomiting, constipation 3. hypertension
3.	Tricyclic antidepressants	xerostomia, constipation, urinary retention, nausea arrhythmia, hypotension, hypertension, tachycardia, myocardial infarction, stroke blurred vision, dizziness, tremor, anxiety, confusion, hallucination
4.	Typical antipsychotics	tachycardia, Hypotension headache, dizziness, fatigue, anxiety, syncope tardive dyskinesia, tardive dystonia, extrapyramidal reactions xerostomia
5.	Atypical antipsychotics	xerostomia, constipation, increased appetite, diarrhea insomnia, agitation, anxiety, dizziness, headache extrapyramidal symptoms, dystonia, hypertonia, motor restlessness
6.	Benzodiazepines	xerostomia, constipation, diarrhea, nausea, vomiting sedation, dizziness, vertigo, nervousness, confusion, headache tachycardia, palpitations, hypotension
7.	Gingko biloba	Antiplatelet effect, anticoagulant effect, increased bleed time

Effects of anti-cognitive disorder drugs on oral health

The action of the Choline esterase inhibitors (ChEIs) are generalized to the brain and hence has some cholinergic effects such as excess saliva secretion, increased peristaltic movement and relaxation of vascular muscles. Patients with AD are advised to take ChEIs and due to the effects of ChEIs, these patients are most likely experience sialorrhea. They also experience increased nausea, vomiting, abdominal pain, and diarrhea [24,25]. This problem of excess salivation possess a great problem during dental treatment in maintaining a dry working field and the retention of the removable prostheses is also compromised due to the modification in the consistency of the saliva [26]

The use of adjunct medications such as Antidepressants, benzodiazepines, and antipsychotics in AD condition has a severe adverse effect on oral cavity . Proper case history and drug history has to be taken in people under these medications as signs and symptoms can vary from xerostomia to hypersalivation. The features of xerostomia are due to the anticholinergic activity. There are many features which occur in the oral cavity due to reduced salivation or no salivation such as tendency of the oral mucosa and lips to become dry, normal oral flora count is altered, flushing of the tissue is decreased leading to loss of buffering capacity, increased plaque formation, due to increased plaque formation there are possibilities of gingivitis, periodontal disease, dental caries, risk of sialadenitis, greater frequency of oral candidiasis.

Other common side effects which are seen are orofacial movement disorders such as involuntary Jaw movements due to effect of antipsychotics and antidepressant medication. Haloperidol has a risk of orofacial tardive dyskinesia, resulting in attrition and fracture of the tooth, breakage of the denture prostheses pain in the orofacial region, degeneration of TMJ, tongue and cheek ulcers ulcers secondary to tongue and cheek biting, difficulty in articulation of speech, difficulty in swallowing, less intake of food ,weight loss and compromised facial esthetics [27].

Drug interactions of anti-cognitive disorder drugs with dental drugs

Patients with AD and dementia related medications have increased susceptibility for adverse drug interactions. Complete past medical history of the patient and drugs being used has to be checked prior to administration of any anesthetics antimicrobials, analgesics. Interactions between medications and adjuncts used in the treatment of AD and dentistry are listed out in Table 4.

**Table 4:** Adverse drug interaction associated with anti-Alzheimer drugs

S.NO	Anti- Alzheimer drugs	Dental drugs	Drug interactions	Dental recommendations
1	Acetylcholinesterase inhibitor: Donepezil Galantamine Rivastigmine	Clarithromycin Erythromycin Itraconazole Ketoconazole	The cholinergic activities are incresed thereby inhibiting the metabolism of donepezil and galantamine	Consult with physician before prescribing prolonged course of medications
		NSAIDs	Increased potential for gastrointestinal irritation and bleeding	Patient’s physician has to be consulted prior using this drug .
2	N-methyl-D-aspartate receptor antagonists: Memantine	Clarithromycin Erythromycin Itraconazole Ketoconazole	Inhibits the drug metabolism of medications which are metabolised via cytochrome P450 liver miocrosomal enzymes	Close monitoring of the patient has to be done when prescribing the drug.
3	Gingko biloba	Aspirin, NSAIDs	Tendency for haemorrhage and spontaneous bleeding as there is platelet aggregation	Use of caution and consultation with physician .

## Conclusions

The pathophysiology of Alzheimer's disease and dementia-related problems are complex involving the cognitive functioning of an individual. Patients with AD and dementia are under various drugs to manage their condition and, hence, it is the duty of a dental specialist or an oral health care provider to understand the patient and their medications. They need to understand the modifications to be done in the medications to provide safe and effective oral health care.
